# Superhydrophobic/Superoleophilic PDMS/SiO_2_ Aerogel Fabric Gathering Device for Self-Driven Collection of Floating Viscous Oil

**DOI:** 10.3390/gels9050405

**Published:** 2023-05-12

**Authors:** Feng Liu, Xin Di, Xiaohan Sun, Xin Wang, Tinghan Yang, Meng Wang, Jian Li, Chengyu Wang, Yudong Li

**Affiliations:** 1 Key Laboratory of Bio-Based Material Science & Technology of Ministry of Education, Northeast Forestry University, Harbin 150040, China; 2 State Key Laboratory of Urban Water Resource and Environment, School of Environment, Harbin Institute of Technology, Harbin 150001, China

**Keywords:** oil collection, oil–water separation, superhydrophobic, superoleophilic

## Abstract

The persistent challenge of removing viscous oil on water surfaces continues to pose a major concern and requires immediate attention. Here, a novel solution has been introduced in the form of a superhydrophobic/superoleophilic PDMS/SiO_2_ aerogel fabric gathering device (SFGD). The SFGD is based on the adhesive and kinematic viscosity properties of oil, enabling self-driven collection of floating oil on the water surface. The SFGD is able to spontaneously capture the floating oil, selectively filter it, and sustainably collect it into its porous fabric interior through the synergistic effects of surface tension, gravity, and liquid pressure. This eliminates the need for auxiliary operations such as pumping, pouring, or squeezing. The SFGD demonstrates exceptional average recovery efficiencies of 94% for oils with viscosities ranging from 10 to 1000 mPa·s at room temperature, including dimethylsilicone oil, soybean oil, and machine oil. With its facile design, ease of fabrication, high recovery efficiency, excellent reclaiming capabilities, and scalability for multiple oil mixtures, the SFGD represents a significant advancement in the separation of immiscible oil/water mixtures of various viscosities and brings the separation process one step closer to practical application.

## 1. Introduction

With the development of the social economy and industrialization, oil pollution such as waste edible oil, mechanical abandoned oil, and industrial spilled oil increases rapidly, which results in a serious threat to the ecosystem and human health [1,2]. Conventional methods and technologies such as flotation, separators, centrifugation, oil containment booms, and skimmers have been developed for oil removal but are not effective for totally eliminating oil from water, especially oil with viscosity, thus making the separation incomplete with oil residual in water [3,4]. Moreover, these methods usually involve low selectivity, tedious operations, energy-consuming processes, low separation efficiency, and so on, which severely blocks the practical usage of these approaches. Therefore, novel materials with special selectivity, good mechanical stability, excellent separation efficiency, and reliable recyclability are urgently needed for the separation of oil/water mixtures [5,6].

Recently, various separation materials with special wettability such as membrane films, porous materials, and gelation have been widely developed [7,8,9,10,11,12]. Superhydrophilic materials with hierarchical structures and water-binding affinity could adsorb water and make water become trapped in the rough structures once contacting water during the separation process [13,14]. The adsorbed water forms a hydration layer, which definitely reduces the contact area between the oil and sample surface and thus decreases the oil adhesive property. During the separation process, these materials could easily attract and filter water content from oil/water mixtures under gravity or external force, often displaying outstanding oil/water separation efficiency [15,16]. However, besides the drawbacks of the rigorous fabrication process, complicated operation steps, and the process being energy-consuming, having low recyclability, and sometimes requiring pump assistance, the large-scale usage of these materials for oil/water separation still remains limited because of their separation style: the water content of the viscous-oil/water mixtures passes through the materials instead of gathering floating viscous oil content from the mixtures, requiring oil/water mixtures to be gathered first [17]. Meanwhile, most reported materials are mainly focused on organic solvents or light oils (e.g., gasoline and diesel) and are still easily polluted and fouled by sticky oils [18,19,20].

Porous absorbent materials with water repellency such as sponges, foam materials, rubber, carbon-based materials, and chemosynthesis adsorbents have drawn much attention to dealing with floating oil assigned to their prominent adsorption characteristics and extrusion property [21,22,23]. The materials could spontaneously and selectively adsorb oil from the water surface due to their lipophilicity and water resistance, usually possessing excellent adsorption capacity and exceptional separation efficiency. Polydimethylsiloxane (PDMS) sorbent has been widely used for oil–water separation due to its high selectivity and recovery rates for oil types, as well as its ease of use and cost-effectiveness compared to other sample preparation methods [24,25,26]. However, its use also has several disadvantages, including higher costs than other sample preparation materials, environmental impact due to the difficulty of biodegradation of the synthetic material, the need for reusability, and limited capacity for large sample volumes. Graphene/PDMS sponge has gained attention as a promising material for oil–water separation due to its high surface area and selective affinity for different types of oil. In addition, its reusability and durability make it a cost-effective solution for oil–water separation [27,28,29]. However, the synthesis and preparation of graphene/PDMS sponge can be challenging and requires advanced methods, resulting in high production costs. Furthermore, the stability of the material may not be consistent over time, leading to degradation and decreased efficiency in oil–water separation. However, it is of concern that these adsorbent materials, once used for viscous oil adsorption and removal, would show a dramatic decline in adsorption properties, leading to an inevitable decrease in recovery efficiencies after a limited number of uses [21]. The primary reason for the decline is due to the stickiness and kinematic viscosity of the oils, which would lead to accumulated contaminants, severe pore fouling, and an irreversible decrease in their adsorption properties. In the meantime, artificial squeezing or pump-driven procedures may require tedious separation operations and high energy consumption. Therefore, novel devices and methods with easy operation, self-driven property, good oil recovery, and stable recyclability should be designed for floating viscous oil collection and removal.

Herein, a superhydrophobic/superoleophilic PDMS/SiO_2_ aerogel fabric gathering device (SFGD) is designed for collection and removal of floating viscous oil spills. Firstly, a layer of superhydrophobic/superoleophilic PDMS/SiO_2_ composite aerogel coating is prepared on the surface of the burlap fabric using PDMS and nano-silica aerogel particles [30,31]. Subsequently, the burlap fabric is combined with porous plastic balls (i.e., by filling the porous plastic balls as an internal support frame inside the burlap sack) to form the SFGD. The device could spontaneously gather floating oil on a water surface and sustainably collect it into a porous sack relying on the effects of surface tension, gravity, and liquid pressure, requiring no auxiliary operations (e.g., pumping, pouring, and squeezing). During the separation process, the partially submerged fabric surface remains superhydrophobic even underwater, which mainly results from the adhered waterproof viscous oil, hierarchical surface structures, and the intrinsic water repellency of the textile surface. Therefore, an interesting phenomenon appears that the water tightly wraps the submerged part of the sack surface while being totally forbidden to pass through the textured surface; simultaneously, the oil could easily flow into the sack while being inevitably locked into the sack by the wrapped water. Finally, the floating viscous oil is collected into a container and the SGFD could be reutilized for the oil/water separation, thus demanding no clean-up treatment. Moreover, the fabrics display good water repellence, excellent wear resistance detected by an oscillating abrasion tester, and prominent reusability via a recycling experiment. The facile prepared SFGD with scalable fabrication, high recovery efficiency, and prominent reclamation for the separation of immiscible oil/water mixtures of various oil viscosities utilizing a self-driven approach is displayed to illustrate the necessity of the unique device designed herein.

## 2. Results and Discussion

### 2.1. Mechanism and Surface Wettability

**Figure 1:**
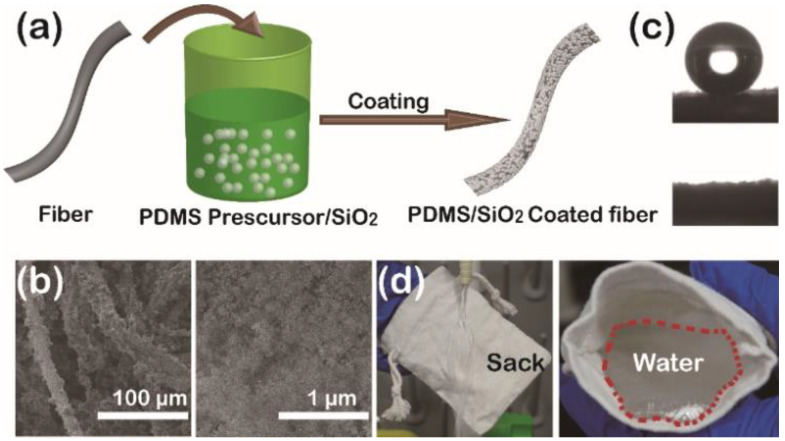
(**a**) Schematic illustration of the construction process for the PDMS/SiO_2_-coated burlap fabric. (**b**) SEM images of the PDMS/SiO_2_ composite aerogel coating on burlap fibers (left) with partial magnification (right). (**c**) Photographs of water contact angle (top) and oil contact angle (bottom) on the coated fabric surface in air conditions, respectively. (**d**) Photographs exhibiting the surface hydrophobicity of the water impacting test (left) and the water holding test (right).

Polydimethylsiloxane has been widely applied for the construction of superhydrophobic/superoleophilic surfaces due to its low surface energy and adhesive properties [32].Nano-silica aerogel particles were prepared by the sol-gel method and used as the surface coating composition for providing additional nanoscale surface roughness and enhancing surface [30]. As shown in Figure 1a, PDMS prepolymer was dissolved into n-hexane solution under stirring and then the added nano-silica aerogel particles were dispersed under ultrasonic treatment because of the aggregation of the particles. After a simple dip-coating procedure and a subsequent drying process, the burlap fabric was coated with a uniform superhydrophobic PDMS/SiO_2_ composite aerogel coating as exhibited in the SEM images (Figure 1b). During the preparation of the PDMS/SiO_2_ composite aerogel coating on the fabric surface, the adhesive properties of the PDMS played a crucial role for both the fabric substrate and silica. The strong adhesion of the PDMS ensured firm and continuous bonding between the coating and the fabric surface, which was essential for the long-term stability and durability of the superhydrophobic coating. The adhesive properties of the PDMS also prevented the coating from peeling or flaking off the fabric substrate, ensuring that the coating remained intact even under high stress or wear conditions. In addition, the addition of nano-silica aerogel particles not only built a rough structure for the overall superhydrophobic coating, but also increased its mechanical stability and durability. The adhesive properties of the PDMS also helped to bond the silica particles to the fabric, forming a strong and uniform coating that could resist wear and tear. Therefore, the combination of the PDMS and silica enhanced the overall performance of the coating and made it more superhydrophobic. With the synergistic effect of hybridization and composite formation between PDMS and nano-silica aerogel particles, a layer of superhydrophobic/superoleophilic PDMS/SiO_2_ composite aerogel coating is formed on the surface of burlap fabric.

Generally, surfaces with low surface energy showed a stronger affinity to oil than water [33,34]. The wettability of the coated burlap fabric was measured in air conditions with water contact angles (WCAs) of 156° (Figure 1c top) and an oil contact angle of 0° (Figure 1c bottom), respectively, showing excellent water repellency and superoleophilicity. Once it contacted the as-prepared sample, the oil droplet would spread on the modified surface (see the Supplementary Information, Figure S2). As demonstrated in Figure 1d, the sack coated by PDMS/SiO_2_ composite aerogel coating displayed excellent water impact resistance (Figure 1d left) and outstanding water-holding properties (Figure 1d right).

**Figure 2:**
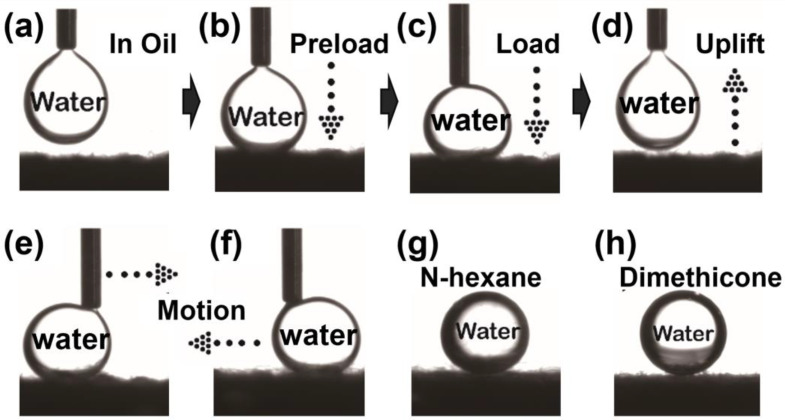
Photographs of (**a**–**f**) the underoil water-adhesion detection process on PDMS/SiO_2_-coated fabric surface and (**g**,**h**) water contact angles on the prepared surfaces under n-hexane and under dimethyl silicone oil, respectively.

As reported, the modified Young’s equation could be not only applicable to analyze the wettability of an oil droplet on a solid surface underwater but also valid to a water droplet on a surface in oil [35,36]. The modified formula of water contact angle on an ideal smooth surface underoil ($\theta_{WO}$) is displayed in Equation (1). According to the equation,

(1) $$\cos\theta_{WO}^{\prime }=r\cos\theta_{WO}$$

where $\theta_{WO}^{\prime }$ represents the water contact angle of a water droplet on the rough surface and *r* is the roughness of the surface. the value of surface roughness (*r*) is greater than 1, illustrating that for material with underoil WCA ($\theta_{WO}$) more than 90°, the real value of $\theta_{WO}^{\prime }$ increase with the strengthening of surface roughness. Thus, the nano-silica aerogel-particles play an essential role in the construction of underoil superhydrophobicity. The wettability of the as-prepared rough surfaces with superhydrophobicity was explored via a contact angle meter (Figure 2 and Figure S3).

In oil, the superoleophilic PDMS/SiO_2_ composite aerogel coating could quickly absorb oil and the oil could be tightly adhered to the textured surface and trapped in the rough structures, which was attributed to the excellent surface affinity to oil. The oil-coated fabric surface demonstrated remarkable superhydrophobicity with a WCA of 154.1° under n-hexane (Figure 2g). As shown in Figure 2a–d, when a water droplet came into contact with the oil/solid composite interface (Figure 2b) under n-hexane and was pressed downward (Figure 2c), the droplet was compressed but did not wet the interface, retaining its intact shape. When the water droplet was lifted, it remained non-adherent to the interface (Figure 2d). Additionally, when the water droplet was brought into contact with the interface and pressed down a certain distance, upon moving the droplet to the right (Figure 2e) or left (Figure 2f), there was no lag or adhesion between the droplet and the interface. Furthermore, as shown in Figure 2h, the WCA on the textured surface under dimethylsilicone oil was 165.8°, providing a solid theoretical foundation for the collection and removal of viscous oils (the WCAs on the composite surface in the other viscous oils can be seen in Figure S3). In summary, the burlap fabric coated by PDMS/SiO_2_ composite aerogel coating possesses predominantly excellent oil affinity and underoil water repellency.

### 2.2. Surface Chemical Component Analysis

**Figure 3:**
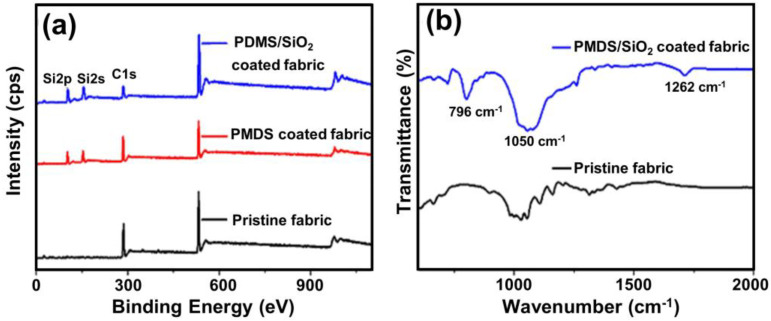
(**a**) XPS survey spectra of pristine burlap fabric, burlap fabric coated with PDMS only, and PDMS/SiO_2_ composite aerogel coating. (**b**) FTIR spectra of pristine burlap fabric and burlap fabric coated with PDMS/SiO_2_ composite aerogel coating.

As displayed in Figure 3, the chemical composition of the prepared burlap fabric was examined by X-ray photoelectron spectroscopy (XPS) (Figure 3a) and FT-IR spectroscopy (Figure 3b).

Table S1 summarizes the quantitative data obtained for pristine burlap fabric, burlap fabric coated with PDMS only, and PDMS/SiO_2_ composite aerogel coating. The XPS graphic of the burlap fabric exhibited C1s, O1s, Si2p, and Si2s peaks as shown in Figure 3a. Comparing the results, there was a significant increase in Si peaks after the fabrics were coated with PDMS and PDMS/SiO_2_. Meanwhile, there was an apparent relatively higher ratio of O to C (43.3/24.1) or Si to C (32.6/24.1) content on the PDMS/SiO_2_-coated fabric compared to the PDMS coated fabric (O to C = 27.6/48.8; Si to C = 23.6/48.8), which could be attributed to the nano-silica aerogel particles [37]. The contents increase could also be qualified through EDX as shown in Figure S4. Figure 3b showed that the absorption peaks at 1050 cm^−1^ and 1262 cm^−1^ were assigned to Si-O-Si stretching vibrations attributed to silicon dioxide and silicon rubber. The occurrence of prominent bands at 796 cm^−1^ represented the Si-C vibrations conforming that the PDMS adhered to the fabric surface. In summary, Figure 3 demonstrated that silicon dioxide and silicon rubber were deposited on the superhydrophobic burlap fabric surface.

### 2.3. Mechanical Robustness

**Figure 4:**
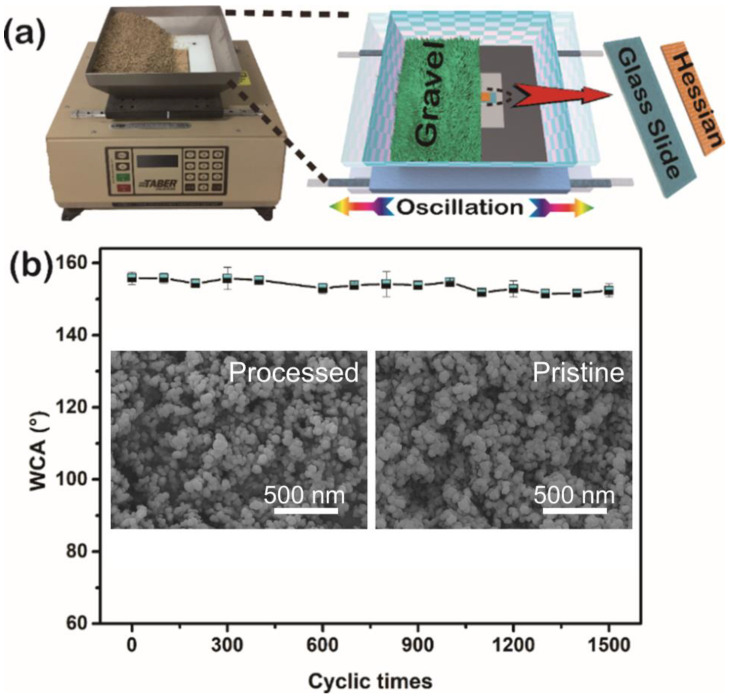
(**a**) Schematic Illustration of surface mechanical detection by an oscillating abrasion tester. (**b**) WCAs change of the burlap fabric by the PDMS/SiO_2_ layer in the process of the abrasion test, and the SEM image after 1500 cycles and the corresponding insert SEM images of the pristine sample and the treated sample.

The stability of micro- and nano-scale rough structures is an essential part of preparing durable special wettability surfaces which deeply affects the practicability of the materials [38]. The stickiness of the PDMS could coat and fix the nanoparticles on the fabric surfaces and the elasticity of the dried PDMS will disperse the forces once subjected to mechanical forces, which could protect the coated nano silica from facing the external force directly. To confirm the mechanical stability of the PDMS/SiO_2_ composite aerogel coating on the fabric surface, the as-prepared burlap fabric was investigated by an oscillating abrasion tester as shown in Figure 4a. The fabric side of the sample was placed into the oscillating abrasion tester equipment with grit covering the whole sample. As exhibited in Figure 4b, the WCA of the sample remained above 152° even after 1500 abrasion cycles showing excellent durability. The stable hydrophobicity of the burlap fabric was primarily attributed to the remaining PDMS/SiO_2_ rough structures (Figure 4b inserted images). Moreover, a blue-dyed water droplet could easily roll off the fabric surface after being treated for 1500 cycles (see the supplementary Information, Figure S6).

### 2.4. Separation of Viscous Oil/Water Mixture

**Figure 5:**
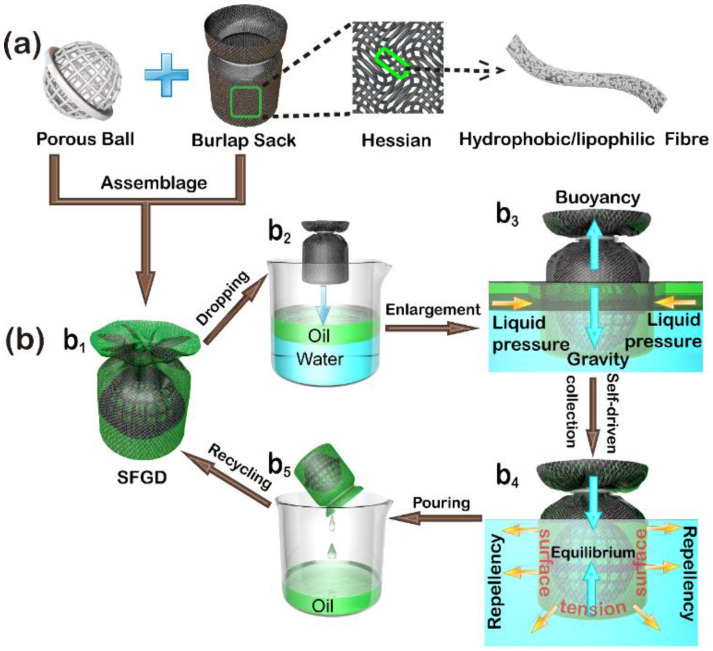
Scheme for (**a**) assembling the superhydrophobic/superoleophilic aerogel fabric gathering device (SFGD) and (**b**) the process (**b_1_**–**b_5_**) of separating the immiscible viscous oil/water mixture.

For understanding the separation mechanism of the immiscible oil/water mixture via the SFGD, a schematic illustration for viscous oil collection and removal was provided as displayed in Figure 5. The SFGD was assembled by a superhydrophobic/superoleophilic burlap sack and a porous plastic ball (Figure 5(a,b_1_)). The porous hollow ball was selected as an internal prop that could keep the sack owning a steady inner space. When dropping into a beaker containing viscous oil/water mixtures (Figure 5(b_2_)), the SFGD would partially submerge in water due to gravity but not sink because of the buoyancy meaning that the average density of the device is lower than water. Subsequently, due to the superhydrophobic/superoleophilic properties of the SFGD surface fabric, the viscous oil on the water surface rapidly wetted the SFGD surface and slowly and self-drivenly passed through the outer fabric of the SFGD under the joint gravity of the SFGD and the oil as well as the liquid pressure, while the water was selectively blocked on the outer side due to the superhydrophobicity of the burlap fabric and the superhydrophobicity under the oil, finally completing the collection of the viscous oil on the water surface (Figure 5(b_3_)). Thus, an interesting phenomenon occurs that the water tightly wraps the submerged part of the sack surface while being totally prevented from penetrating the textured surface; however, the oil could be easily attracted and flow into the sack while being inevitably locked into the sack by the wrapped water. As shown in Figure 5(b_4_), when equilibrium (zero resultant force) was achieved, the oil-filled SFGD still partially submerged in water and remained unsinkable ascribed to its lower density than the water, and it maintained water repellency because of the water resistance of the collected oil and surface superhydrophobicity. Subsequently, the device was taken out and the oil was poured into a container. The leakage rate of the collected oil was rather low due to the relatively high kinematic viscosity of the inherent oil property, which could guarantee the oil recovery. Finally, after the pouring process, the SFGD was directly reused for viscous oil collection and removal without any wash treatment (Figure 5(b_1_,b_5_)).

**Figure 6:**
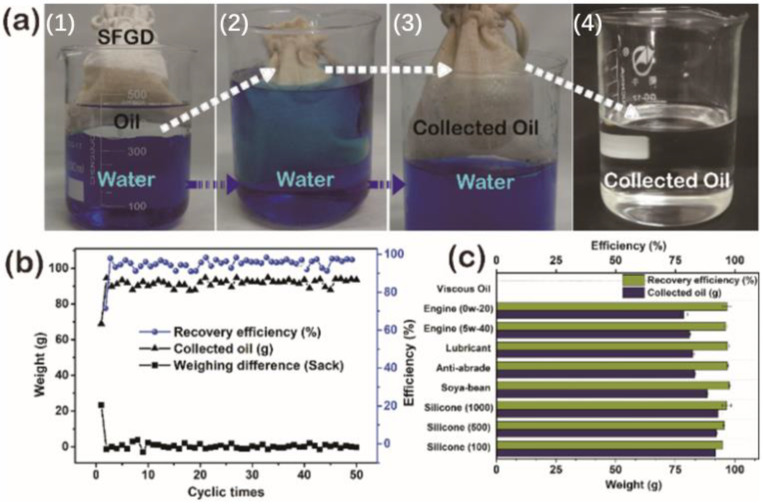
(**a**) Photographs for viscous oil removal from the water surface by SFGD. (**b**) The recovery of viscous silicone oil and the weighing difference of the sack before and after usage. (**c**) Recovery capacity and recovery efficiency of various kinds of viscous oils.

Figure 6a shows that floating silicon oil (100 ± 8 mPa·s) was collected and removed from the blue-dyed water surface via the SFGD following the steps in Figure 5b. It required 2 h for managing the oil collection. Obviously, on first use, the SFGD was gradually adsorbed with silicon oil during the gathering process which led to the mass increase of the oil-poured empty SFGD. Therefore, after the oil removal process, the weight of the uncleaned empty SFGD increased from 14.67 g to 38.09 g, and the weight difference of the device before and after usage was 23.42 g as shown in Figure 6b. The mass and recover efficiency of the collected oil was 68.9 g and 71.5%, respectively. However, when repeating the above separation via an uncleaned device, the weight difference of the SFGD could be controlled to ±2 g with the average recovery of 94.81% after 50 cycles (Figure 6b). Furthermore, during the long-time water resistance detection (see the Supplementary Information, Figure S6), the SFGD filled with the collected oil remained floating on the water for 15 days with an oil recovery efficiency of no less than 95.8%, showing excellent practicability. The SFGD could be applied to collect various viscous oils, such as dimethylsilicone oil (viscosity 100 ± 8 mPa·s, 500 ± 8 mPa·s, 1000 ± 8 m mPa·s), soybean oil, lubricant oil, anti-wear hydraulic oil, and gasoline engine oil, with recovery efficiency above 94% as exhibited in Figure 6c. 

**Figure 7:**
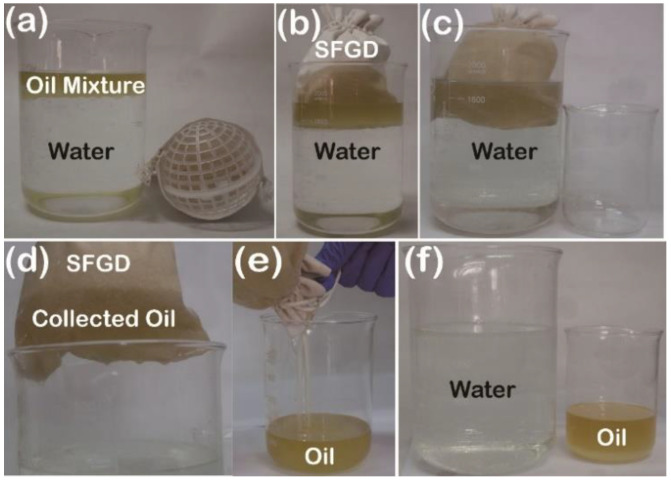
Photographs for the collection process of an oil mixture from the water surface via SFGD. (**a**) SFGD, oil mixture, and water. (**b**–**e**) Oil collection process. (**f**) Cleaned water and recovered oil.

To evaluate the practical usage of the device (burlap sack: 15 × 20 cm, porous plastic ball diameter: 8 cm), 300 mL of viscous oil mixture containing dimethylsilicone oil (viscosity 100 ± 8 mPa·s), soybean oil, lubricant oil, anti-wear hydraulic oil, and gasoline engine oil at the ratio of 1:1:1:1:1 was added to in a 2000 mL beaker filled with 1500 mL of water (Figure 7a). The special wettability of the fabric provided the key foundation of the self-driven, gravity and liquid pressure aided, floating viscous oil collection device as illustrated. When dropped into the beaker, the enlarged SFGD with oil binding affinity was gradually wetted and adhered to by the oil mixture as shown in Figure 7b,c. The floating viscous oil mixture was driven to be filtered by the fabric surface and collected in the inner surface of the sack (Figure 7d,e) under the effectiveness of the surface tension, gravity, and liquid pressure and simultaneously. Though repelled by the hydrophobic fabric, the water seamlessly wrapped the device due to liquid pressure and indeed prevented the oil mixture from leaking from the sack. When taken out, the device could retain the oil mixture without any leakage, which mainly resulted from the adhesive properties and kinematic viscosity of the oil. Thus, the oil mixture was completely collected and removed from water (Figure 7f), exhibiting that this device with remarkable functionality could be easily scalable. Thus, the self-driven oil collection SFGD was successfully applied for the separation and removal of immiscible viscous oil/water mixtures with excellent recyclability and practicability.

## 3. Conclusions

In summary, the burlap fabric coated by a layer of superhydrophobic/superoleophilic PDMS/SiO_2_ composite aerogel coating were initially and creatively utilized for self-driven floating viscous oil collection. The SFGD could spontaneously attract, filter, and collect the floating oils under the synergetic effect of surface tension, gravity, and liquid pressure, requiring no extra operations such as pumping, oil/water mixture collecting, and squeezing. The superhydrophobic/superoleophilic burlap sack with excellent mechanical robustness and functionality could be easily scaled by a flexible dip-coating method and utilized for oil/water separation, displaying excellent oil recovery efficiency even after 50 cycles of usage. Furthermore, during long-time water resistance detection, the SFGD filled with the collected oil remained floating on water for 15 days with oil recovery efficiency no less than 95.8%, showing excellent endurance and practicability. It is worth stating that the SFGD could be scaled for the efficient elimination of large multiple oil mixtures on water, demonstrating the versatility for oil removal. We firmly believe that such a smartly designed oil-collection system could provide a unique perspective for dealing with floating viscous oil pollution.

## 4. Materials and Methods

### 4.1. Materials

Burlap sack (length and width: 10 cm × 12 cm and 15 cm × 20 cm) and porous suspended plastic ball (diameter: 6 mm and 8 mm) were supplied by Alibaba. PDMS was provided by Dow Corning Co., Ltd (Sylgard 184, Midland, MI, USA)). Tetraethoxysilane (TEOS, chemically pure), and NH_3_·H_2_O (28%) were obtained from Tianjin Kaitong Chemical Reagent Co, Ltd (Tianjin, China). Dimethylsilicone oil (viscosity 100 ± 8 mPa·s), dimethylsilicone oil (viscosity 500 ± 8 mPa·s), and dimethylsilicone oil (viscosity 1000 ± 8 mPa·s) were purchased from Shanghai Aladdin Biochemical Technology Co., Ltd (Shanghai, China). Methylene blue, ethanol, and n-hexane were provided by Sinopharm Chemical Reagents Co., Ltd. (Shanghai, China). Soybean oil was procured from a local market. Lubricant oil, anti-wear hydraulic oil, gasoline engine oil (0W-20), and gasoline engine oil (5W-40) were obtained from Petro-China Co., Ltd. (Beijing, China). All chemicals were used as received without further purification.

### 4.2. Synthesis of Superhydrophobic and Superoleophilic Burlap Sack

Firstly, the nano-silica aerogel particles were prepared with an alkali-base catalyzed sol–gel method by drying under ambient pressure. In brief, ethanol (90 mL), TEOS (10 mL) and deionized water (10 mL) were mixed and stirred to prepare a mixture solution, and then 2 mL NH_3_·H_2_O used as the catalyst was added dropwise into the mixture at room temperature under magnetic stirring (400 rpm) for 0.5 h. The gelation process usually takes around 12 h to complete, and the gels are subsequently allowed to age for 72 h in order to enhance the gel network. N-hexane was used to replace the solvent of wet gel. After rinsing with n-hexane 3–4 times, the wet gel was dried under ambient pressure to obtain the bulk silica aerogel. The bulk silica aerogel was ground into silica aerogel particles.

Secondly, a colloidal solution composed of PDMS and nano-silica aerogel particles was prepared. The colloidal solution was prepared by sonicating 90 mL of n-hexane, 3 g of PDMS prepolymer (Sylgard 184A), 0.3 g of PDMS prepolymer (Sylgard 184B) and 3.6 g of nano-silica aerogel particles for 20 min using an ultrasonic disruptor, followed by magnetic stirring for 10 min.

Finally, the burlap sack was totally washed with deionized water, n-hexane, and ethanol under ultrasonic treatment for 20 min, respectively. Then, the burlap fabric was immersed into the colloidal solution. Subsequently, the immersed fabric sack was taken out, rinsed with n-hexane, and dried at 60 °C for 3 h. The resulting burlap fabric was coated with a layer of superhydrophobic/superoleophilic PDMS/SiO_2_ composite aerogel coating that adheres uniformly to its surface.

### 4.3. Characterization

The morphological surfaces of the samples were observed by a scanning electron microscope (SEM, FEI QUANTA 200, Hillsboro, OR, USA) operating at 15 kV. The surface chemistry composition was detected by Fourier transform infrared spectroscopy (FT-IR, Thermos Fisher Scientific, Nicolet 6700, Waltham, MA, USA) and X-ray photoelectron spectroscopy (XPS, ESCALAB 250Xi, Thermo Fischer Scientific, Waltham, MA, USA). The contact angles were measured with a 5 μL deionized water droplet or an oil droplet at room temperature using an optical contact angle meter (OCA20 system, DataPhysics Instruments GmbH, Filderstadt, Germany), and the final contact angle was determined by averaging the measurements taken from at least five different positions on samples which were adhered onto the glass slide by double-sided adhesive tape.

### 4.4. Stability Test

Mechanical stability superhydrophobic/superoleophilic PDMS/SiO_2_ composite aero-gel coating on surface of burlap fabric was assessed by an oscillating abrasion tester and recycling experiment for viscous oil/water separation. In order to be easily measured by a contact angle meter, the as-prepared waterproof fabric was cut into pieces (1 cm × 3 cm) before adhering one of them onto a glass slide (2.5 cm × 7.6 cm) that was sticky on the central area of a square board (10 cm × 10 cm) by double-sided adhesive tape as well. Then, the fabric side of the above sample was placed into the oscillating abrasion tester equipment with 800 mL of grit covering the whole sample. During the oscillation process, the rolling grit constantly abraded the textile surface at a speed of 130 cycles/min. The oscillating process was repeated for a certain number of times to detect the stability of superhydrophobicity. The structural stability of superhydrophobic/superoleophilic composite aerogel coating was assessed by cycling experiments of viscous oil/water mixture separation.

### 4.5. Separation of the Immiscible Viscous Oil/Water Mixture

Typically, an immiscible viscous oil/water mixture consisting of 300 mL water dyed by methylene blue and 100 mL of silicone oil (viscosity 100 ± 8 mPa·s) was prepared and served in a beaker container. Then, the SFGD was put into the beaker container for 3 h for viscous oil gathering and removal. The SFGD could be directly reused to collect the floating viscous oil without any clean-up process. Meanwhile, the fabric device could be applied to collect various kinds of daily used viscous oil such as soybean oil, vacuum pump oil, anti-wear hydraulic oil, and gasoline engine oil (5W-40) following the same separation process. 

The oil recovery efficiency was defined as *W* (%) and calculated by Equation (2):

(2) $$W=\frac{m_{1}}{m_{0}}\times 100\%\;$$

where $m_{0}$ and $m_{1}$ were the mass of the oil before and after the separation process, respectively.

In this study, the average mass of the oil-coated SFGD was 37.61 g and the density of the silicone oil was 0.963 g/mL. The calculated theoretical value of the maximum volume of the collected oil under certain conditions was calculated as 1016.5 mL when reaching equilibrium (see the Supplementary Information, Figure S1), exhibiting that the collected volume totally covered the volume of the SFGD containing a porous plastic ball with a volume about 113 mL. Therefore, 100 mL of viscous oil was selected for the demonstrative experiments of oil collection and removal.

## Data Availability

Not applicable.

## Supplementary Materials

The following supporting information can be downloaded at: https://www.mdpi.com/article/10.3390/gels9050405/s1. Figure S1: (**a**) Force equilibrium analysis diagram of the oil-filled and partially submerged SFGD and (**b**) the corresponding photograph. Figure S2: Photographs of an oil droplet quickly spread on the surface in the air once contacting the superoleophilic fabric surface. Figure S3: Underoil WCAs of various oils. Figure S4: EDX elemental analysis images of (**a**) PDMS coated fabric and (**b**) PDMS/SiO_2_ layer coated fabric. Figure S5: Photo images of the blue-dyed water droplet rolling off the fabric surface after being treated by an oscillating abrasion tester for 1500 cycles. Figure S6: (**a**) Photographs of the SFGD dropped into a beaker containing oil/water mixture, (**b**) the oil-filled SFGD partially submerged in water for 15 days, (**c**) photographs of the oil-filled SFGD taken out of the container, and (**d**) the volume of the collected oil. Table S1: Surface relative composition of XPS analysis for pristine burlap fabric, burlap fabric coated with PDMS only and PDMS/SiO_2_ layers.
