# Supplementary material for: Superhydrophobic/Superoleophilic PDMS/SiO2 Aerogel Fabric Gathering Device for Self-Driven Collection of Floating Viscous Oil
Source: Gels. 2023 May 12;9(5):405. doi: 10.3390/gels9050405 (PMC10217856; doi:10.3390/gels9050405)
Supplement: Supplementary file 1 [file gels-09-00405-s001.zip › gels-2197988-supplementary.pdf]

Superhydrophobic/Superoleophilic PDMS/SiO2 Aerogel Fabric Gathering Device for Self-Driven Collection of Floating Viscous Oil

Feng Liu [1†], Xin Di [1†], Xiaohan Sun [1], Xin Wang [1], Tinghan Yang [1], Meng Wang [2], Jian Li [1], Chengyu Wang *[1] and Yudong Li *[1]

[1] Key Laboratory of Bio-based Material Science & Technology of Ministry of Education, Northeast Forestry University, Harbin 150040, China

[2] State Key Laboratory of Urban Water Resource and Environment, School of Environment, Harbin Institute of Technology, Harbin 150001, China

* Correspondence: wangcy@nefu.edu.cn (C. W.); lydlnn0000@163.com (Y. L.)

† These authors contribute equally to this work.

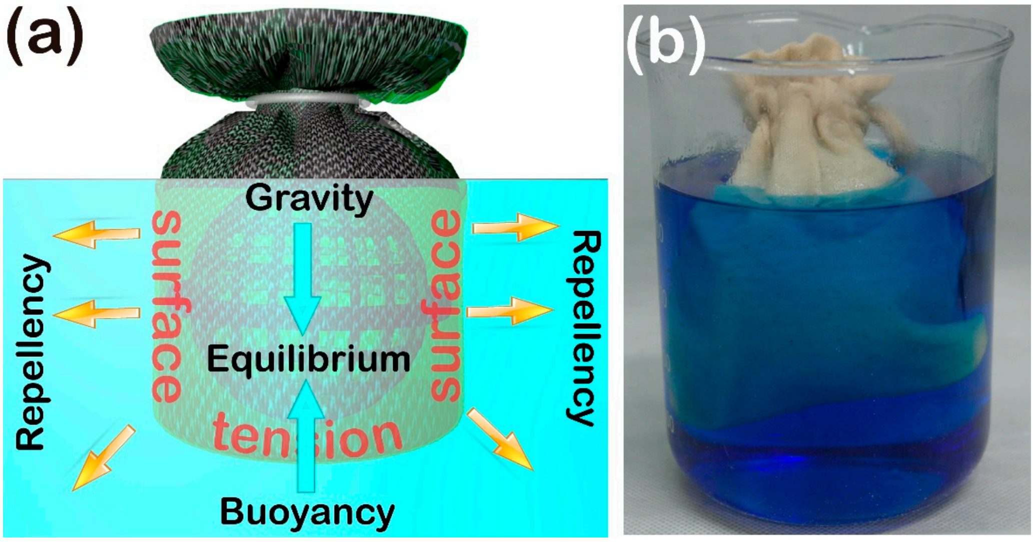

Figure S1: (a) Force equilibrium analysis diagram of the oil-filled and partially-submerged SFGD and (b) the corresponding photograph.

The theoretical basis at equilibrium was shown as below.

$$G_{SFGD} = F_{buoyancy} - G_{oil} = \rho_H g V_H - \rho_o V_o g \quad \text{(S1)}$$

$$G_{SFGD} = (m_1 + m_2 + m_3)g \quad \text{(S2)}$$

$$G_{oil} = \rho_o V_o g \quad \text{(S3)}$$

$$F_{buoyancy} = \rho_H g V_H \quad \text{(S4)}$$

where G_{SFGD}, m_1, m_2, and m_3 represents the gravity of SFGD, the quality of the

collected oil, the quality of the coated oil on sack surface, respectively (Equation (S2)); G_{oil}, ρ_o, V_o represents the gravity of the collected oil, the density of the oil, and the volume of the collected oil, respectively (Equation (S3)); $F_{buoyancy}$, ρ_H, and V_H represents the buoyancy resulting from water, the density of water and the repelled volume of water, respectively (Equation (S4)).

Assuming that $V_o = V_H = V$ at equilibrium (Figure S6b4), thus the Equation (S2) was modified as V=G_{SFGD} / [(ρ_H -ρ_o) g]. In this study, the mass of the average oil-coated SFGD was 37.61g and the density of the silicone oil was 0.963 g/mL. According to the modified Equation (S2), the calculated V was 1016.5 mL exhibited that the theoretical maximum volume of the collected oil and totally covered the volume of the SFGD containing a porous plastic ball with oil volume about 113 mL. Therefore, 100 mL of viscous oil was selected for the demonstrative experiments of oil collection and removal.

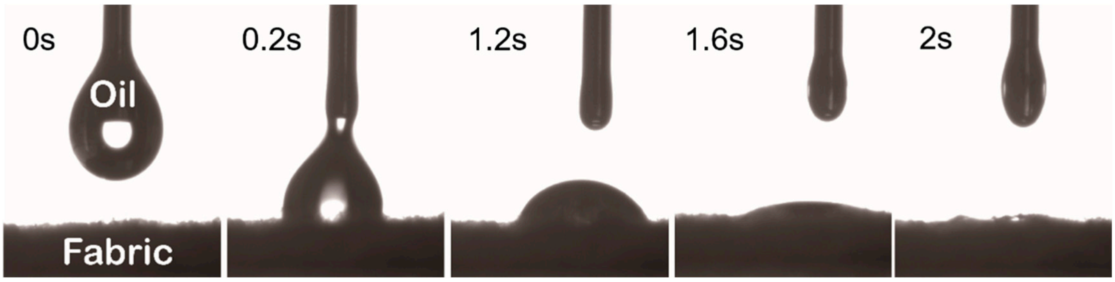

Figure S2: Photographs of an oil droplet quickly spread on the surface in the air once contacting the superoleophilic burlap fabric surface.

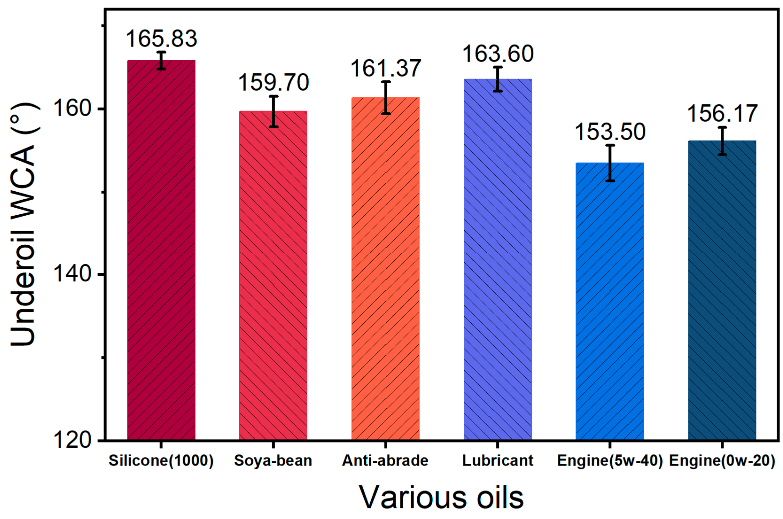

Figure S3: Underoil WCAs of various oils.

As reported, the modified Young's equation could be not only applicable to analyze the wettability of an oil droplet on a solid surface underwater but also valid to a water droplet on a surface in oil [1,2]. According to Young's equation in different three-phase interface systems (solid/water/air, oil/water/air and solid/water/oil), the water contact angle on an ideal smooth surface underoil (θ_{WO}) could be modified as below:

$$\cos\theta_{WO} = \frac{\gamma_{WV}\cos\theta_W - \gamma_{OV}\cos\theta_O}{\gamma_{WO}} \quad \text{(S5)}$$

Where θ_W and θ_O represent the water contact angle and oil contact angle in air, respectively, γ_{WV}, γ_{OV} and γ_{WO} are the interfacial tension of water/air, oil/air, and water/oil, respectively. In this study, the value of the WCA (θ_W) of superhydrophobic/superoleophilic burlap sack is more than 90° and the value of the OCA (θ_O) is less than 90° ,concluding that the water contact angle (θ_{WO}) of superhydrophobic/superoleophilic burlap sack is greater than 90° . Obviously, in reality, surfaces are most likely to be uneven with rough structures. Therefore, in order to solve this apparent issue, the equation underoil could also be derived as below in

comparison to the Wenzel and Cassie equations for air.

$$\cos\theta'_{WO} = r\cos\theta_{WO} \tag{S6}$$

Where θ'_{WO} represents the water contact angle of a water droplet on the rough surface, and r is the roughness of the surface. According to the Equation (S6), the value of r is greater than 1, illustrating that for material with WCA (θ_{WO}) more than 90°, the real value of θ'_{WO} will increase with the strengthening of surface roughness. Figure S3 showed the WCAs under various oil conditions.

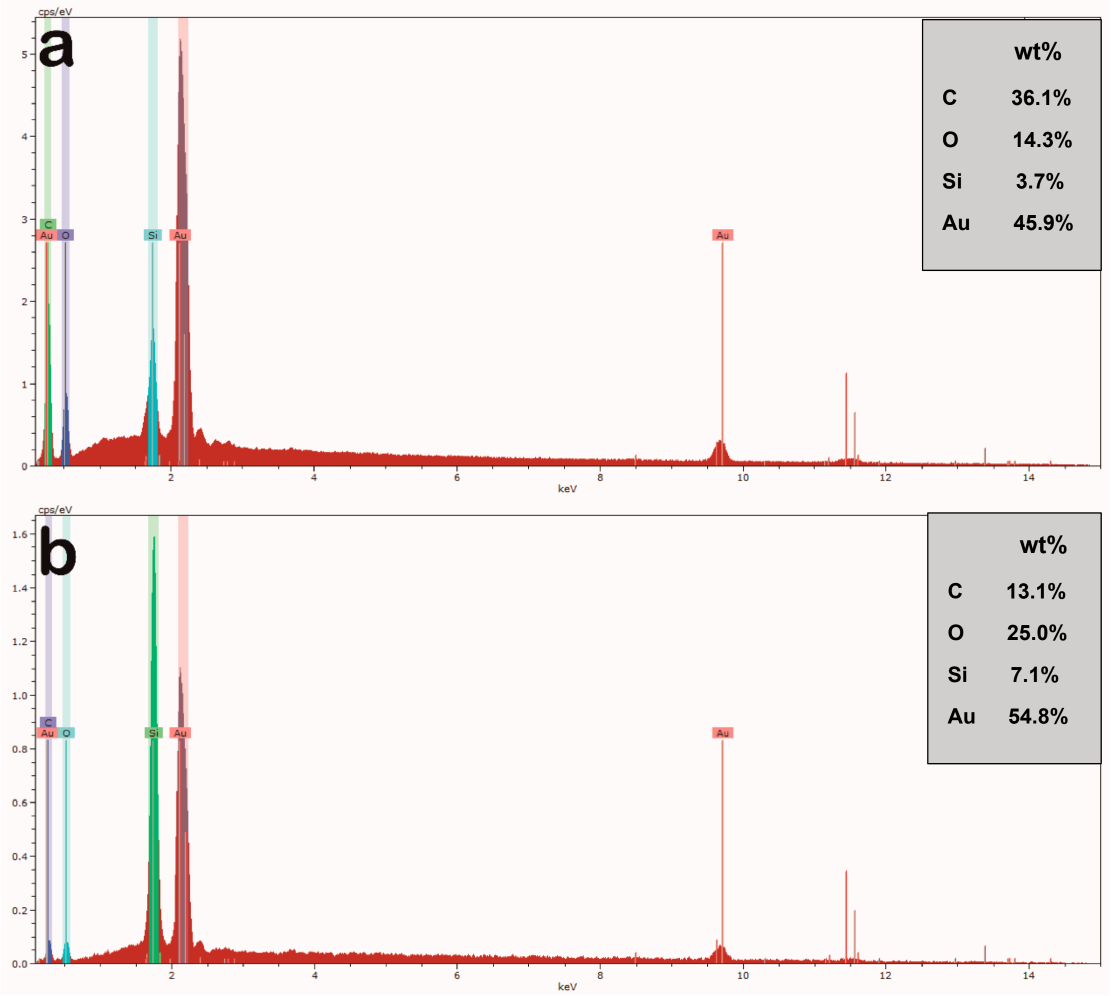

Figure S4: EDX elemental analysis images of (a) PDMS coated fabric and (b) PDMS/SiO_2 layer coated fabric.

In comparison to PDMS coated samples, the ratio of O to C or Si to C contents on the PDMS/SiO_2 fabric surface showed an obvious increase.

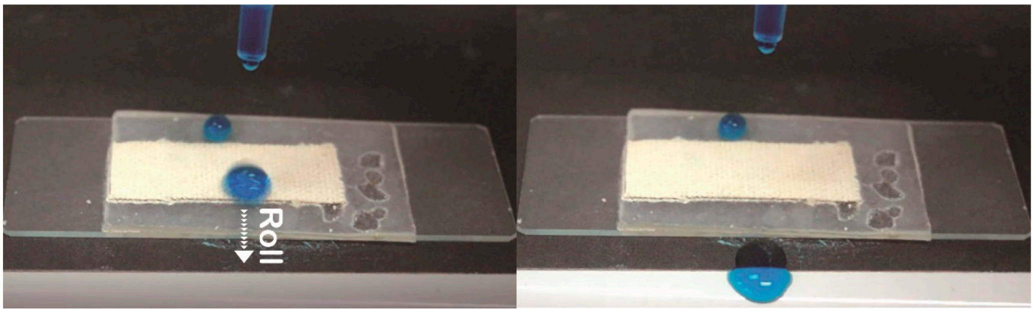

Figure S5: Photo images of blue-dyed water droplet rolling off the fabric surface after being treated by an oscillating abrasion tester for 1500 cycles.

The grits damaged the double-sided adhesive surface with grooves appearing, showing excellent abrasion resistance of the fabric structure.

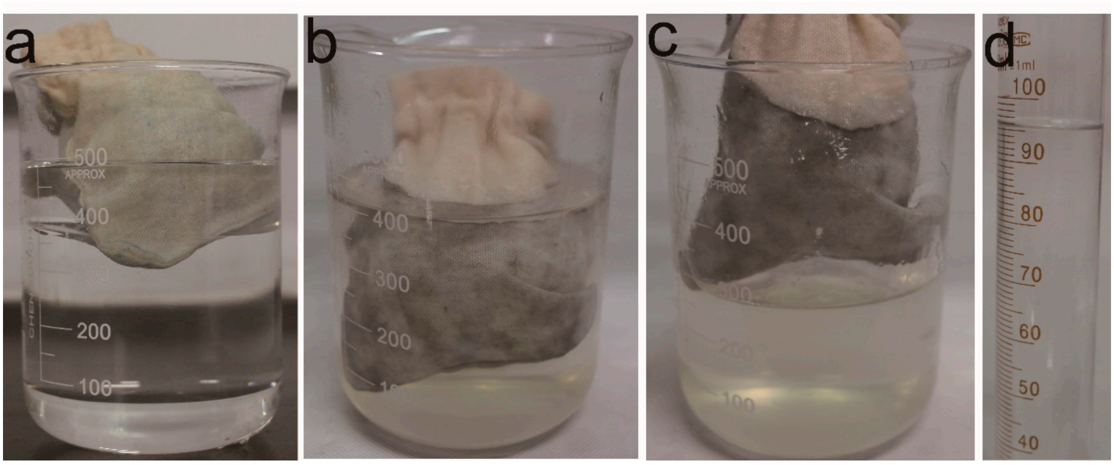

Figure S6: (a) Photographs of SFGD dropped into a beaker containing oil/water mixture, (b) the oil-filled SFGD partially submerged in water for 15 days, (c) Photographs of the oil-filled SFGD taken out of the container and (d) the volume of the collected oil.

After 15 days of immersion in water, the sack surface became moldy while the collected oil changed slightly with recovery efficiency remaining high (Figure S6d). By comparison between Figure S6a and Figure S6c, the liquid level of water dropped significantly, which mainly due to the evaporation of water in an unsealed container.

Table S1 Surface relative composition of XPS analysis for pristine burlap fabric, burlap fabric coated with PDMS only and PDMS/SiO_2 layers.

Sample	%C	%Si	%O
Pristine burlap fabric	42.1	0.1	57.8
burlap fabric coated with PDMS layer	48.8	23.6	27.6
burlap fabric coated with PDMS/SiO_2 layer	24.1	32.6	43.3

References

1. Liu, M.; Wang, S.; Wei, Z.; Song, Y.; Jiang, L. Bioinspired Design of a Superoleophobic and Low Adhesive Water/Solid Interface. *Advanced Materials* **2009**, *21*, 665-669, doi:10.1002/adma.200801782.
2. Chen, C.; Weng, D.; Mahmood, A.; Chen, S.; Wang, J. Separation Mechanism and Construction of Surfaces with Special Wettability for Oil/Water Separation. *ACS Appl Mater Interfaces* **2019**, *11*, 11006-11027, doi:10.1021/acsami.9b01293.
